# Recent progress in the racemic and enantioselective synthesis of monofluoroalkene-based dipeptide isosteres

**DOI:** 10.3762/bjoc.13.262

**Published:** 2017-12-12

**Authors:** Myriam Drouin, Jean-François Paquin

**Affiliations:** 1 Département de chimie, Université Laval, 1045 avenue de la Médecine, Pavillon Alexandre-Vachon, Québec (Québec) G1V 0A6, Canada

**Keywords:** dipeptide isosteres, monofluoroalkene-based amide bonds, monofluoroalkenes, peptides, synthesis

## Abstract

Monofluoroalkenes are fluorinated motifs that can be used to replace amide bonds. In order to be incorporated into peptides, it is normally necessary to first synthesize a dipeptide where the amide bond has been replaced with a monofluoroalkene. In that context, this review will present the racemic and enantioselective synthesis of monofluoroalkene-based dipeptide isosteres described since 2007. Some applications of those compounds will also be presented.

## Introduction

Nowadays, the pharmaceutical industry is interested in the development of new categories of drugs. While small molecules were the principal targets in the last decades [1], larger biomolecules, such as peptides, are now widely studied [2–3]. The interest of these biopolymers originates, in part, from their high potency and selectivity towards the target, which results in a decrease of the toxicity and/or side effects. However, peptides have a poor metabolic stability [2].

**Figure 1:**
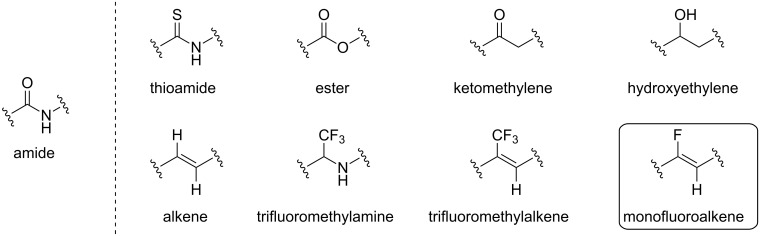
Selected amide bond isosteres.

A solution to enhance the stability of peptides is to modify their structure, in particular the amide bond linkage. Different moieties can be used as amide bond isosteres and some are illustrated in Figure 1 [4–6].

**Figure 2:**
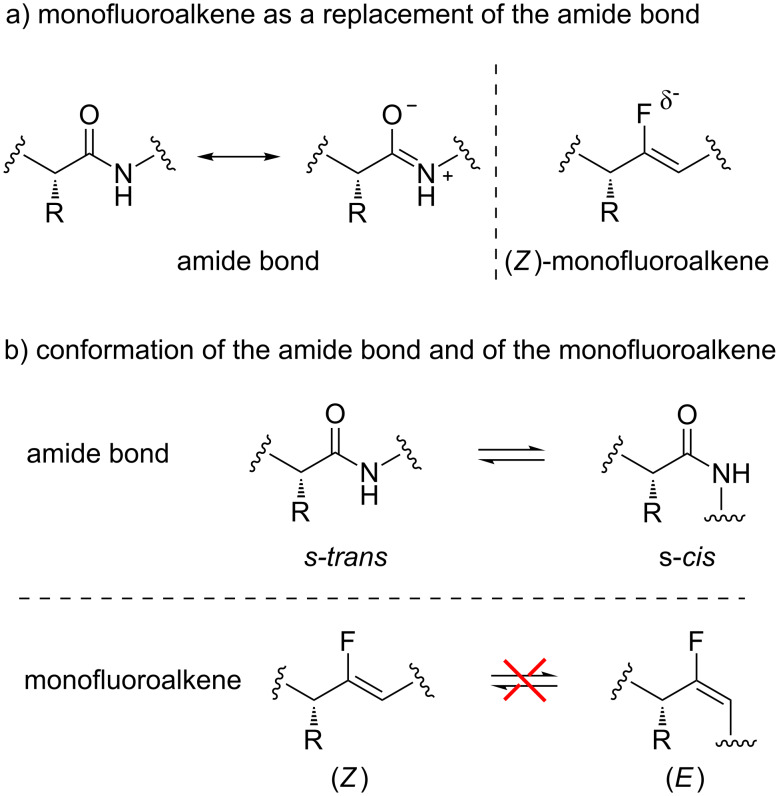
Monofluoroalkene as an amide bond isostere.

Of those amide bond isosteres, the monofluoroalkene is of particular interest as it possesses many relevant characteristics (Figure 2a). The resonance in the amide generates a double bond character between the carbon of the carbonyl and the nitrogen, which is responsible of the slow rotation around this bond. Furthermore, the negative charge is located on the oxygen atom and the dipole moment of the amide bond is 3.6 D [7]. The amide bond can also perform hydrogen bonds, with the oxygen atom as the hydrogen bond acceptor and N–H as hydrogen bond donor. This characteristic is important for the formation of secondary structures and folding into tertiary and quaternary structures. To have a good amide bond isostere, these different aspects should be reproduced, which is mostly the case with the monofluoroalkene moiety [8]. The monofluoroalkene is a rigid molecule as it possesses a double bond. Furthermore, the fluorine atom bears a partial negative charge, with a dipole moment of 1.4 D. Finally, the monofluoroalkene has the ability to accept a hydrogen bond through the fluorine atom [9]. Geometrically, the monofluoroalkene is quite similar to the amide bond. The C=O bond of the amide is 1.228 Å, compared to 1.376 Å for the C–F bond, and the C–N bond is 1.368 Å compared to 1.333 Å for the C=C bond [5,10–12]. Also, the amide structure is found in Nature as the s-*trans* or s-*cis* isomer, which can be in equilibrium (Figure 2b) [13]. However, most of the time it is found as the s-*trans* isomer to minimize steric interaction and to favour a linear and less hindered shape [14]. A notable exception is found in the case of the proline, where the s-*cis* isomer is favoured [15]. With the monofluoroalkene moiety, it is possible to mimic selectively one or the other isomer as no equilibrium exists between them. As such, the (*Z*)-monofluoroalkene is an analogue of the s-*trans* amide bond, while the (*E*)-monofluoroalkene mimics the s-*cis* form.

Considering those favourable properties, monofluoroalkenes constitute an interesting amide bond isostere, thus many researches have investigated their synthesis and application [16–22]. In order to be incorporated into peptides, it is normally necessary to first synthesize a dipeptide where the amide bond has been replaced with a monofluoroalkene. This review is the follow-up of the last one published by Taguchi and Yanai in 2009 which covered the literature until 2007 [5] and will discuss the new developments on the racemic and enantioselective synthesis of monofluoroalkene-based dipeptide isosteres from 2008 to September 2017. First, synthetic approaches to analogues in which there is no side chain or where the side chain stereochemistry is not controlled will be highlighted. This will be followed by the presentation of the synthesis of analogues where the side chain stereochemistry is controlled. In both cases, the review will be divided according to the monofluoroalkene-based dipeptide isosteres prepared. Finally, recent applications will be described.

## Review

### Analogues in which there is no side chain or where the side chain stereochemistry is not controlled

#### Gly-ψ[CF=CH]-Gly

**Scheme 1:**
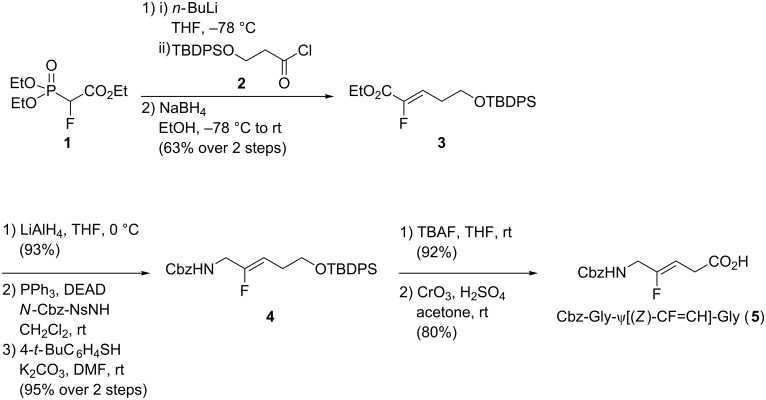
Synthesis of Cbz-Gly-ψ[(Z)-CF=CH]-Gly using a HWE olefination by Sano and co-workers.

The Gly-ψ[CF=CH]-Gly analogue is the simplest one, as it does not present side chains. Its synthesis was performed by two groups, using in both cases an olefination reaction. Sano’s group was interested in the Horner–Wadsworth–Emmons (HWE) olefination to develop the synthesis of α-fluoro-α,β-unsaturated ester **3**, which can be used as a precursor for the synthesis of monofluoroalkene-based dipeptide isosteres [23]. Cbz-Gly-ψ[(*Z*)-CF=CH]-Gly **5** was obtained in seven steps (Scheme 1). First, triethyl 2-fluoro-2-phosphonoacetate (**1**) was converted into the α-fluoro-α,β-unsaturated carbonyl **3** using the HWE olefination. The (*Z*)-isomer was obtained with complete selectivity. Then, reduction of the ester into the corresponding alcohol followed by a Mitsunobu reaction allowed the insertion of the NH-carboxybenzyl moiety to afford **4**. Finally, removal of the *tert*-butyldiphenylsilyl group using *tert*-*n*-butylammonium fluoride, followed by oxidation with the Jones reagent, provided the *C*-terminal carboxylic acid **5**.

**Scheme 2:**
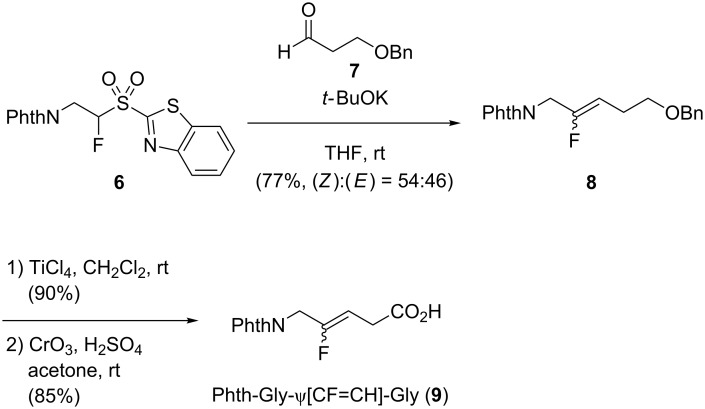
Synthesis of Phth-Gly-ψ[CF=CH]-Gly using the Julia–Kocienski olefination by Lequeux and co-workers.

In 2011, Lequeux and co-workers used rather the Julia–Kocienski olefination to access Phth-Gly-ψ[CF=CH]-Gly **9**, from benzothiazolyl fluoroaminosulfones (Scheme 2) [24–25]. The Julia–Kocienski olefination of 3-alkoxypropanal **7** with phthalimido sulfone **6** afforded the corresponding monofluoroalkene **8** as a (*Z*):(*E*) mixture (54:46). Removal of the benzyl group using titanium tetrachloride gave the free alcohol which was oxidized to provide the N-protected dipeptide isostere **9**. Some limitations were observed towards the compatibility of the N-protecting groups and in particular, *N*-*tert*-butoxycarbonyl-protected amines were not compatible with this methodology.

#### Xaa-ψ[CF=CH]-Gly

**Scheme 3:**
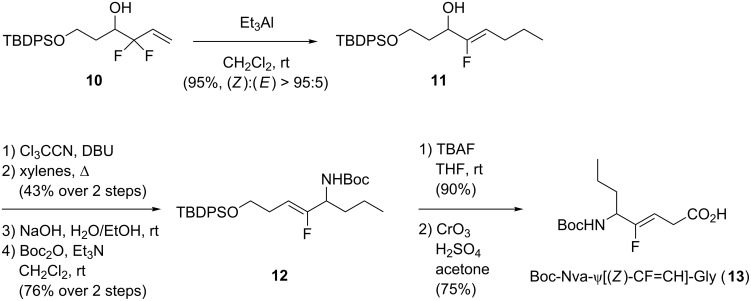
Synthesis of Boc-Nva-ψ[(*Z*)-CF=CH]-Gly by Taguchi and co-workers.

To access Xaa-ψ[CF=CH]-Gly isosteres, a S_N_2’ reaction upon 3,3-difluoropropene substrates can be used, as shown by Taguchi’s group. The synthesis of monofluoroalkenes starting from 3,3-difluoropropenes and using trialkylaluminium reagents was developed. Using this methodology, they were able to prepare Boc-Nva-ψ[CF=CH]-Gly isostere [26] via a S_N_2’ reaction (Scheme 3). The defluorinative allylic alkylation of terminal 3,3-difluoropropene **10** with triethylaluminium selectively provided the corresponding (*Z*)-monofluoroalkene **11**. In this case, the use of Et_3_Al allowed access to a norvaline (Nva) isostere. Then, alcohol **11** was converted into the trichloroimidate, and heating in xylenes permitted a [3,3]-sigmatropic rearrangement. At this stage, the trichloroimidate was transformed into an NHBoc moiety. Deprotection of the alcohol followed by Jones oxidation gave the final dipeptide isostere **13**.

**Figure 3:**
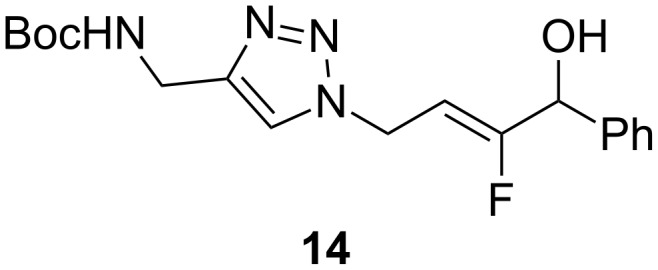
Mutant tripeptide containing two different peptide bond isosteres.

Taguchi and co-workers proposed a variant of the defluorinative reaction using heteroatom nucleophiles using aluminum-based reagents such as Me_2_AlCl and (iPrO)_2_AlN_3_, and (*Z*) selectivity was observed for the formation of the monofluoroalkene [27]. When dimethylaluminum chloride was used, the resulting allylic chloride reacted easily in a S_N_2 reaction to give a more functionalized molecule. For example, treatment of the chlorinated monofluoroalkene with NaN_3_ provided the corresponding N_3_-containing monofluoroalkene. The azide group underwent a 1,3-dipolar cycloaddition to give a 1,2,3-triazole, which is also a peptide bond isostere [6]. Using this strategy, a mutant tripeptide containing two different peptide bond isosteres could be synthesized (Figure 3).

**Scheme 4:**
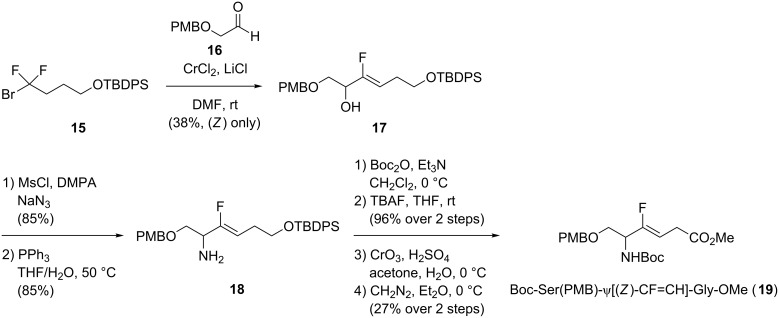
Chromium-mediated synthesis of Boc-Ser(PMB)-ψ[(*Z*)-CF=CH]-Gly-OMe by Konno and co-workers.

In 2016, Konno and co-workers developed a stereoselective chromium-mediated C–F bond cleavage followed by a C–C bond formation to access (*Z*)-monofluoroalkenes with excellent selectivity (Scheme 4) [28]. The chromium-mediated coupling of 1-bromo-4-(*tert*-butyldiphenylsiloxy)-1,1-difluorobutane (**15**) with aldehyde **16** led to the formation of monofluoroalkene **17**. This was then reacted with sodium azide and a further Staudinger reduction gave **18**. Boc protection of the resulting amine **18**, cleavage of the alcohol protecting group, Jones oxidation and formation of the methyl ester afforded the corresponding dipeptide isostere Boc-Ser(PMB)-ψ[(*Z*)-CF=CH]-Gly-OMe (**19**). In the same way, Boc-Val-ψ[(*Z*)-CF=CH]-Gly-OMe, Boc-Leu-ψ[(*Z*)-CF=CH]-Gly-OMe and Boc-Ala-ψ[(*Z*)-CF=CH]-Gly-OMe were prepared.

#### Xaa-ψ[CF=C]-Pro

**Scheme 5:**
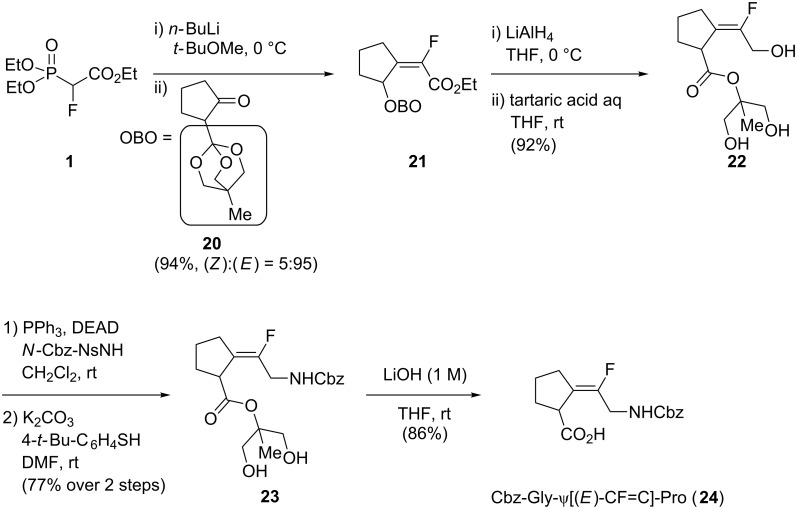
Synthesis of Cbz-Gly-ψ[(*E*)-CF=C]-Pro by Sano and co-workers.

The methodologies presented in the last decades used the olefination of cyclopentanone derivatives as the key step to access Xaa-ψ[CF=C]-Pro, via either a Peterson olefination or a HWE olefination [29–31]. In all cases, the selectivity was modest and the isomers had to be separated by flash chromatography. In 2014, Sano and co-workers reported a selective synthesis of Xaa-ψ[CF=C]-Pro with a new cyclopentanone derivative **20** bearing a bulky 2-(4-methyl-2,6,7-trioxabicyclo[2.2.2]octan-1-yl) group (OBO), which favoured the formation of the (*E*)-isomer in the HWE olefination (Scheme 5). The (*E*)-monofluoroalkene was thus obtained in an excellent selectivity using *n*-butyllithium in *tert*-butyl methyl ether. The resulting ester **21** was reduced using lithium aluminum hydride, and treatment with tartaric acid deprotected the OBO, thus providing the free triol **22**. This was then converted into a protected amino group employing a Mitsunobu reaction. Finally, removal of the nosyl group, followed by hydrolysis using lithium hydroxide, afforded the targeted isostere **24**.

**Scheme 6:**
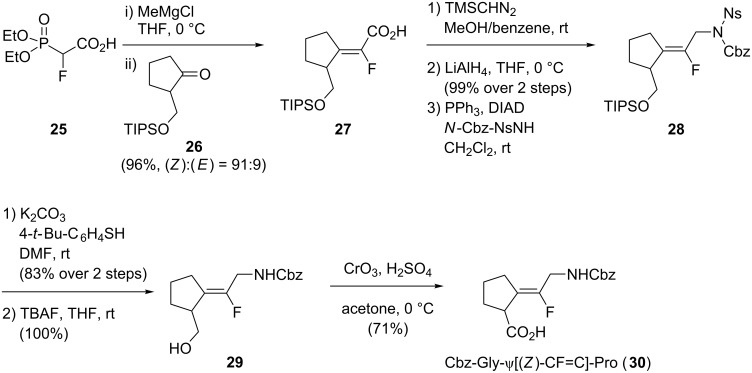
Synthesis of Cbz-Gly-ψ[(*Z*)-CF=C]-Pro by Sano and co-workers.

Sano and co-workers also worked on the Mg(II)-promoted stereoselective synthesis of (*Z*)-monofluoroalkenes (Scheme 6) [32–33]. HWE olefination promoted by Mg(II) of (diethoxyphosphoryl)fluoroacetic acid (**25**) with triisopropylsilyl-protected 2-hydroxymethylcyclopentanone **26** was realized with excellent yield and stereoselectivity. Esterification of the resulting carboxylic acid **27** into the corresponding methyl ester using trimethylsilyldiazomethane, followed by its reduction to the corresponding alcohol and a Mitsunobu reaction, permitted the incorporation of the N*-*terminal moiety. Then, removal of the Ns group of **28** and deprotection of the primary alcohol was performed to obtain **29** which underwent a Jones oxidation to give the final dipeptide isostere **30**.

### Analogues in which the side chain stereochemistry is controlled

#### Gly-ψ[CF=CH]-Xaa

**Scheme 7:**
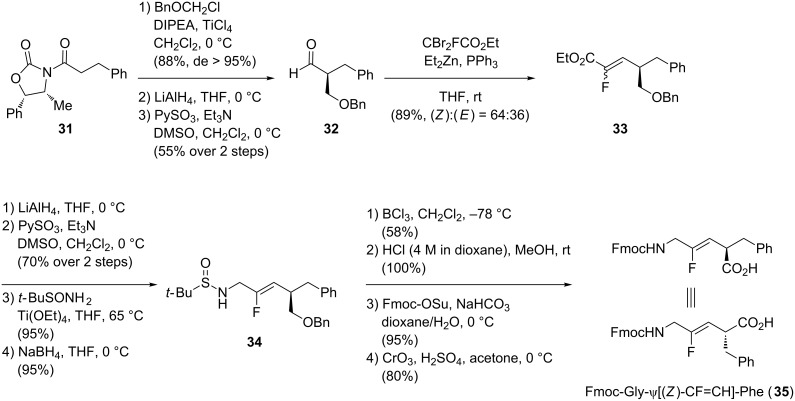
Stereoselective synthesis of Fmoc-Gly-ψ[(*Z*)-CF=CH]-Phe by Pannecoucke and co-workers.

Different strategies have been used over the years to access Gly-ψ[CF=CH]-Xaa isosteres in which the side chain stereochemistry of the Xaa is controlled. This could be achieved using an olefination reaction, a metathesis reaction or a copper-mediated reduction of 3,3-difluoropropenes. Pannecoucke’s group employed a chiral auxiliary, the Evans oxazolidinone, to prepare the non-racemic dipeptide isostere **35** (Scheme 7) [34]. Stereoselective alkoxymethylation on the oxazolidinone derivative **31** was first achieved with an excellent yield (88%) and diastereoselectivity (de > 95%). The chiral auxiliary was then removed and the free alcohol was oxidized to the corresponding aldehyde **32**. Alkene **33** was then obtained after olefination of **32** with low selectivity ((*Z*):(*E*) = 64:36). The resulting ester **33** was then reduced to the corresponding aldehyde, followed by the formation of the terminal imine and its subsequent reduction to access the N-terminal moiety of **34**. The alcohol and the amine deprotections were then achieved, followed by reprotection of the amine with a fluorenylmethyloxycarbonyl group. Oxidation of the remaining alcohol to the corresponding carboxylic acid provided the dipeptide isostere **35**.

**Scheme 8:**
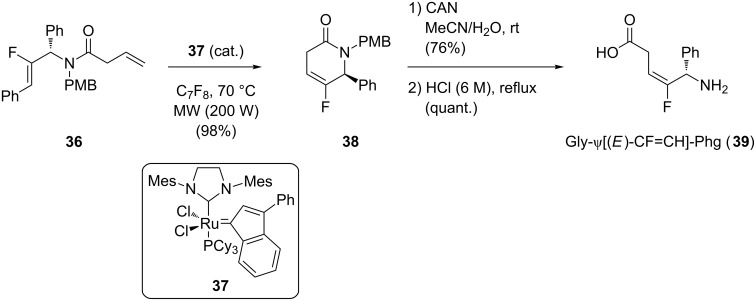
Ring-closure metathesis to prepare Gly-ψ[(*E*)-CF=CH]-Phg by Couve-Bonnaire and co-workers.

Couve-Bonnaire and co-workers developed the preparation of (*E*)-monofluoroalkene dipeptide isosteres towards an intramolecular ring-closure metathesis (Scheme 8) [35]. The bis-alkene **36** underwent a ring-closure metathesis reaction in the presence of catalyst **37** under microwave irradiation to give lactam **38**. Deprotection of the amine followed by acidic opening of the ring gave the (*E*)-monofluoroalkene **39** in good yield. The reaction was also performed on the racemic starting material to confirm that the process did not induce any epimerization. This methodology was then extended to the synthesis Gly-ψ[(*E*)-CF=CH]-Gly (not shown).

**Scheme 9:**
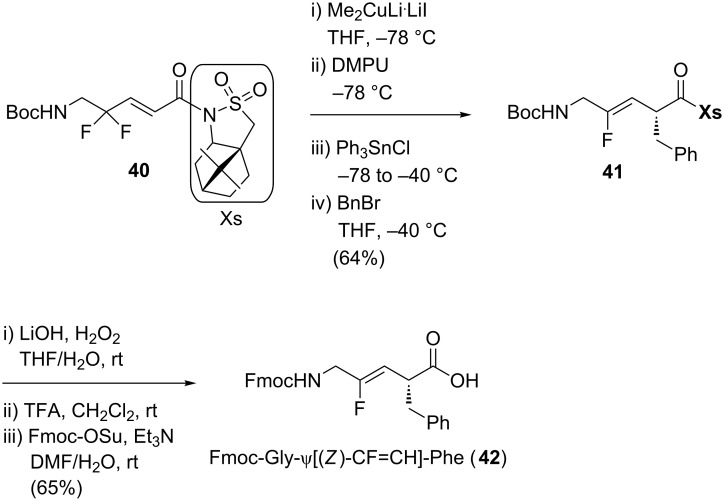
Stereoselective synthesis of Fmoc-Gly-ψ[(*Z*)-CF=CH]-Phe by Dory and co-workers.

Finally, Dory and co-workers reported the synthesis of Fmoc-Gly-ψ[(*Z*)-CF=CH]-Phe (Scheme 9) [36].Their work was inspired by the methodology reported by Fujii, Otaka and co-workers, which showed that the sultam moiety is a useful chiral auxiliary to control the stereochemistry during the incorporation of the lateral chain (see Scheme 15) [37]. The copper-mediated reduction of 3,3-difluoropropene **40** bearing a sultam (Xs) as a chiral auxiliary followed by α-alkylation afforded the monofluoroalkene **41**. Hydrolysis of the chiral auxiliary followed by deprotection of the Boc-protected amine and its subsequent reprotection by a Fmoc group gave the final dipeptide isostere **42**.

#### Xaa-ψ[CF=CH]-Gly

**Scheme 10:**
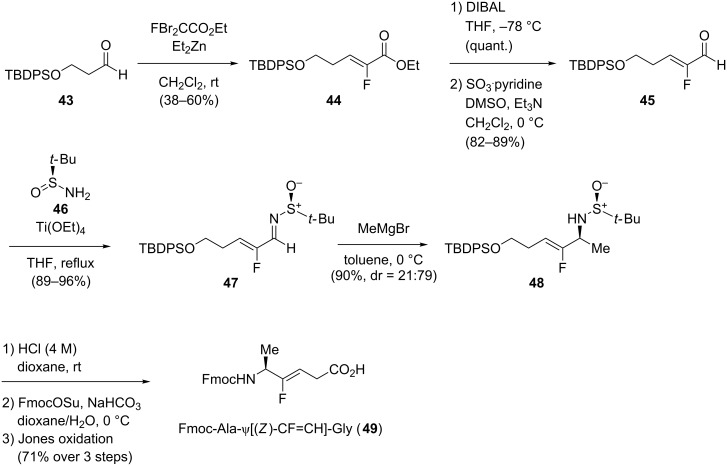
Diastereoselective addition of Grignard reagents to sulfinylamines derived from α-fluoroenals by Pannecoucke and co-workers.

The development of new methodologies to access Xaa-ψ[CF=CH]-Gly isosteres with control of the stereochemistry at the side chain will be discussed in this section. In particular, olefination reaction, defluorinative reduction of 3,3-difluoropropene derivatives and electrophilic fluorination of alkenylstannanes are presented. Pannecoucke’s group described the synthesis of monofluoroalkenes from α-fluoro-α,β-unsaturated aldehydes **45**, which are more easily accessible than the corresponding enones (Scheme 10) [38]. Their synthesis started with the olefination of aldehyde **43** which gave the corresponding monofluoroalkene **44**. Reduction with subsequent oxidation of the ester gave the corresponding aldehyde **45** which was then transformed into the α-fluoroenimine **47**. This was selectively converted into the corresponding sulfinylamines using Grignard reagents to access (*S*)-amino acids **48**, while addition of organolithium reagents gave (*R*)-amino acids. A sequence of N- and O-deprotection, *N*-Fmoc-protection and oxidation to the carboxylic acid afforded the final Fmoc-Ala-ψ[(*Z*)-CF=CH]-Gly (**49**).

Then, it was discovered that the diastereoselectivity of the addition of the Grignard reagent on **47** was enhanced when dimethylzinc (Me_2_Zn) was used as an additive (Table 1) [39]. Indeed, triorganozincates (Me_2_(R)ZnMgX) were formed in situ and these reagents activated favourably the substrates towards the stereoselective addition of the alkyl chain.

**Table 1:** Diastereoselective addition of Grignard reagents to sulfinylamines derived from α-fluoroenals with Me_2_Zn as additive.

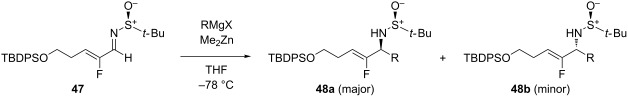			

entry	RMgX	yield (%)	dr

**1**	iPrMgCl	63	93:7
**2**	iBuMgBr	75	98:2
**3**	BnMgCl	96	84:16
**4**	(allyl)MgBr	74	67:33
**5**	(vinyl)MgBr	98	91:9

**Scheme 11:**
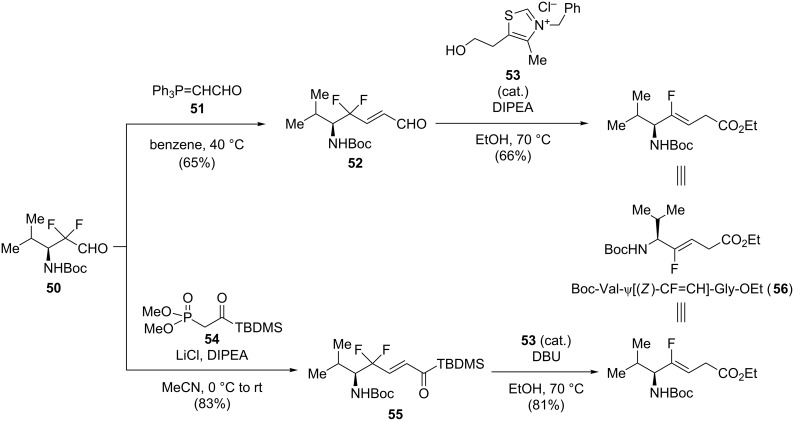
NHC-mediated synthesis of monofluoroalkenes by Otaka and co-workers.

To prepare Boc-Val-ψ[(*Z*)-CF=CH]-Gly-OEt, Otaka’s group developed an intramolecular redox reaction of 3,3-difluoropropenes using N-heterocyclic carbenes (NHCs, Scheme 11) [40]. The reaction was first performed on the γ,γ-difluoro-α,β-enal **52** which was synthesized via a Wittig olefination of **50**. The resulting monofluoroalkene Boc-Val-ψ[(*Z*)-CF=CH]-Gly-OEt was obtained in good yield. Afterwards, the γ,γ-difluoro-α,β-enoylsilane **55**, obtained after HWE olefination using dimethyl phosphonoacylsilane **54**, was found to facilitate the NHC-catalyzed reduction and gave in this way the dipeptide isostere **56** in excellent yield.

**Scheme 12:**
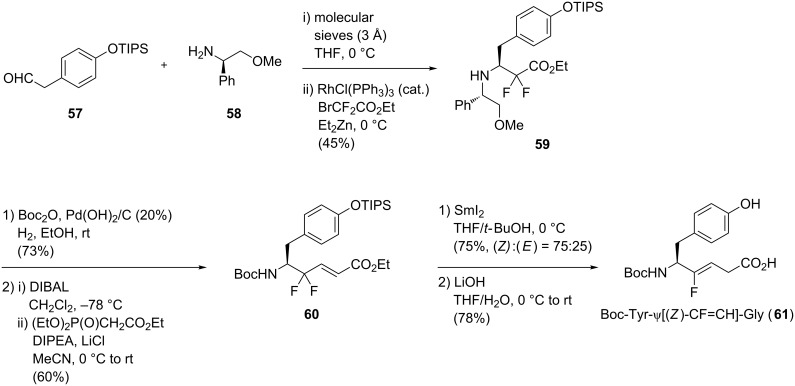
Stereoselective synthesis of Boc-Tyr-ψ[(*Z*)-CF=CH]-Gly by Altman and co-workers.

The defluorinative reduction could also be performed using samarium iodide. Altman and co-workers proposed the synthesis of Boc-Tyr-ψ[(*Z*)-CF=CH]-Gly using a diastereoselective Reformatsky–Honda condensation, a (*E*)-selective HWE olefination and a SmI_2_ reduction as key steps (Scheme 12) [41]. First, Reformatsky–Honda reaction of TIPS-protected phenylacetaldehyde **57** with the chiral auxiliary (*L*)-phenylglycine derivative **58** afforded **59**. Removal of the chiral auxiliary and subsequent Boc protection were then performed. Reduction of the ester followed by HWE olefination of the resulting aldehyde gave the 3,3-difluoropropene **60**. The latter was reduced in the presence of SmI_2_ to furnish the (*Z*)-monofluoroalkene with good yield. A final saponification gave the monofluoroalkene-based dipeptide isostere **61**.

Finally, Fürstner’s group developed the silver-mediated fluorination of functionalized alkenylstannanes to access monofluoroalkenes [42]. Hydrostannation of the N-protected ynamines **62** followed by electrophilic fluorination with Selectfluor gave the corresponding (*Z*)-monofluoroalkenes **64** in good yields (Table 2). The reported results showed that the methodology was suitable to replace an amide bond and could be used in late-stage fluorination to access monofluoroalkene-based dipeptide isosteres.

**Table 2:** Silver-mediated fluorination of functionalized alkenylstannanes.

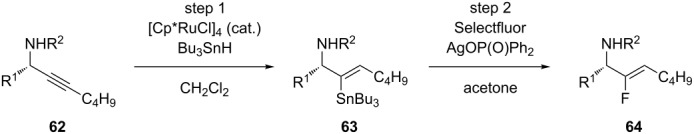			

entry	R^1^	R^2^	yield (step 2, %)

**1**	iBu	Cbz	77
**2**	iBu	P(O)(OPh)_2_	84
**3**	iBu	*t*-BuSO_2_	81
**4**	iBu	Ns	54
**5**	iPr	Cbz	76
**6**	Ph	*t*-BuSO_2_	76
**7**	Ph	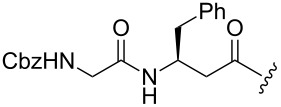	83

#### Xaa-ψ[CF=CH]-Xaa

**Scheme 13:**
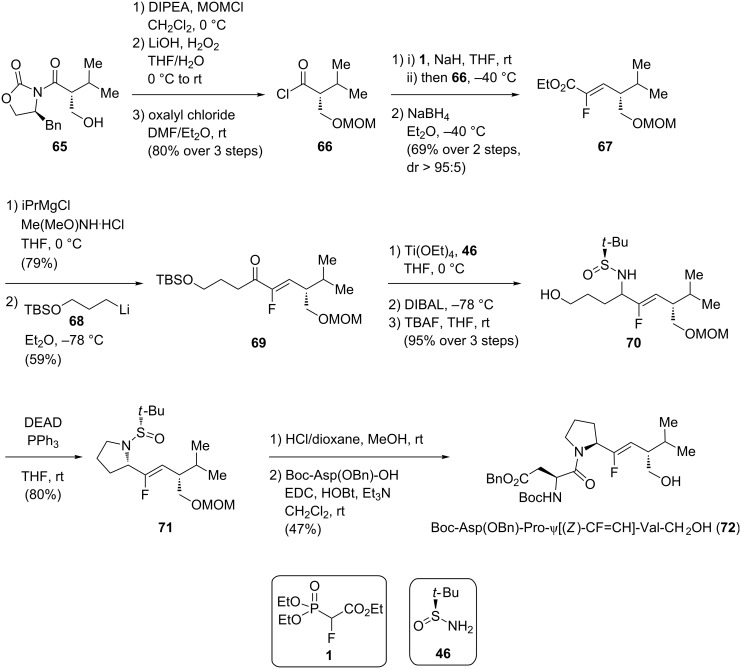
Synthesis of the tripeptide Boc-Asp(OBn)-Pro-ψ[(*Z*)-CF=CH)-Val-CH_2_OH by Miller and co-workers.

The preparation of Xaa-ψ[CF-CH]-Xaa derivatives represents a synthetic challenge, as the stereochemistry of two side chains should be controlled during the synthesis. Here, the monofluoroalkenes can be access either by an olefination reaction or a S_N_2’ reaction starting from 3,3-difluoropropenes. First, Miller reported the asymmetric synthesis of a monofluoroalkene using a chiral auxiliary (Scheme 13) [43]. The synthesis started with the alcohol protection of known compound **65** followed by chiral auxiliary removal and acylation of the resulting carboxylic acid. A HWE olefination was performed in two steps on **66** to give the (*Z*)-monofluoroalkene **67** as a single isomer (dr > 95:5). Conversion of the ester into the corresponding Weinreb amide, followed by addition of an organolithium reagent gave the corresponding ketone **69**. Four further steps gave the ring skeleton for the proline residue of **71**, i.e., formation of the chiral sulfinylimine, reduction into the corresponding sulfinylamine using DIBAL, deprotection of the terminal alcohol and Mitsunobu ring closure into the corresponding pyrrolidine derivative. Then, simultaneous deprotection of the amine and the alcohol in acidic conditions followed by coupling with Boc-Asp(OBn)-OH gave the final tripeptide isostere Boc-Asp(OBn)-Pro-ψ[(*Z*)-CF=CH)-Val-CH_2_OH (**72**).

**Scheme 14:**
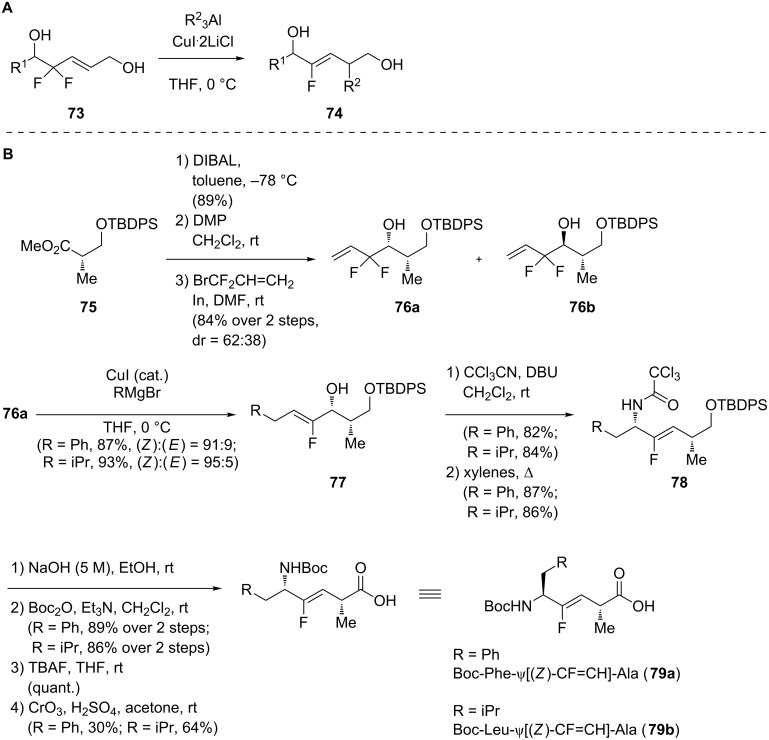
Copper-catalyzed synthesis of monofluoralkenes by Taguchi and co-workers.

In the last years, Taguchi and co-workers described the synthesis of the monofluoroalkenes **74** by S_N_2’ reaction between 4,4-difluoro-5-hydroxyallylic alcohols **73** and Gilman reagent prepared in situ from trialkylaluminium reagents and CuLi (Scheme 14A) [44–45]. Even if the diastereoselectivity of the reaction was excellent, two problems remained. First, an excess of trialkylaluminium reagent and of copper had to be used. Second, trialkylaluminium reagents are not widely available. As an alternative, they proposed in 2011 a new synthetic route using Grignard reagents, which are widely available or can be easily synthetized in the laboratory (Scheme 14B) [46]. Unfortunately, these reagents did not react with the 4,4-difluoro-5-hydroxyallylic alcohols **73**. Terminal 3,3-difluoropropenes **76** were then prepared starting from the commercially available protected chiral hydroxyl ester **75**. Reduction to the aldehyde followed by coupling with bromodifluoropropene gave two diastereoisomers **76a** and **76b** separable by flash chromatography. Then, the copper-catalyzed defluorinative allylic alkylation using Grignard reagents was performed on **76a** and monofluoroalkenes **77** were obtained in good yields and high selectivity. Claisen rearrangement and further modifications (hydrolysis of the trichloroacetoamide, Boc protection of the free amine, deprotection of the alcohol and Jones oxidation to give the carboxylic acid) afforded the final dipeptide isosteres **79a** and **79b**.

**Scheme 15:**
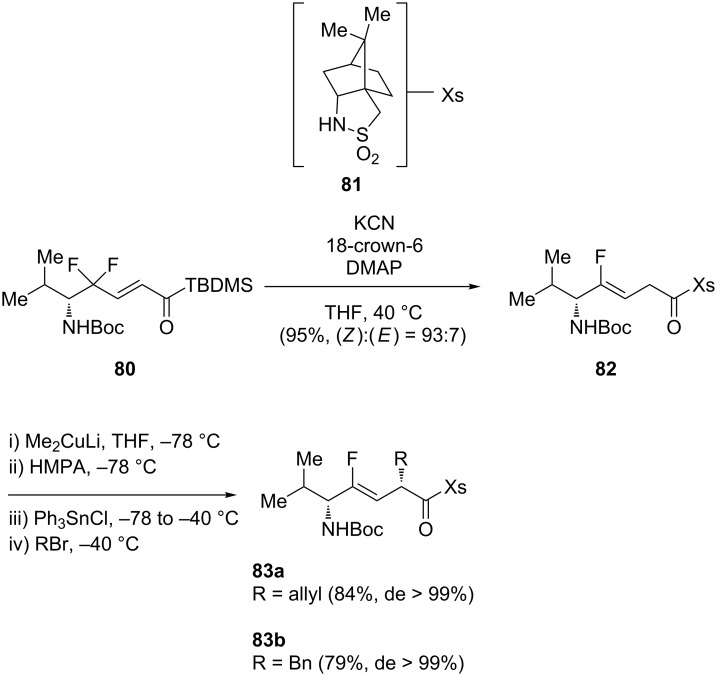
One-pot intramolecular redox reaction to access amide-type isosteres by Otaka and co-workers.

The sultam Xs moiety has also been used as chiral auxiliary for the synthesis of Xaa-[CF=CH]-Xaa [37]. Otaka and co-workers developed a one-pot methodology to access amide isosteres at the *C*-terminal (Scheme 15) [47]. Cyanide-mediated reductive defluorination of γ,γ-difluoro-α,β-enoylsilane **80** in the presence of 18-crown-6 followed by addition of camphorsultam **81** gave the corresponding monofluoroalkene **82**. Then, α-alkylation of the amide with either allyl bromide or benzyl bromide provided the corresponding dipeptide isosteres **83a** and **83b**. Interestingly, different amino acids, such as H_2_N-Gly-OEt, H_2_N-Val-OMe and H_2_N-Pro-OMe, could be used instead of the sultam **81** to access tripeptide isosteres in a racemic manner (not shown).

**Scheme 16:**
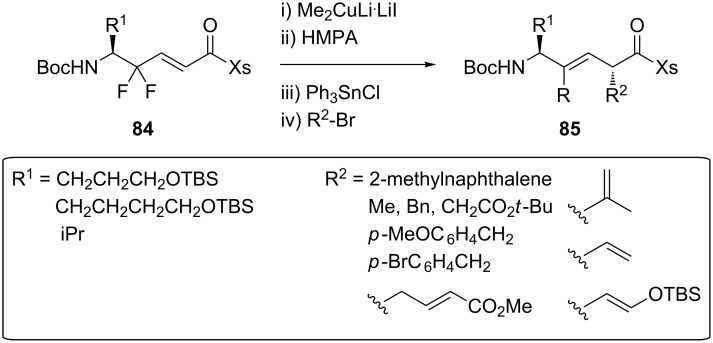
Copper-mediated reduction, transmetalation and asymmetric alkylation by Fujii and co-workers.

Fujii and co-workers also used the sultam Xs as a chiral auxiliary but started their synthesis with 3,3-difluoropropenes bearing a *N*-enoyl sultam moiety **84** instead. A 3-key step strategy involving a copper-mediated reduction, a transmetalation and an asymmetric alkylation was adopted for the preparation of monofluoroalkenes **85** (Scheme 16). After some synthetic modifications, Fmoc-Orn(Ns)-ψ[(*Z*)-CF=CH]-Orn(Ns) [48], Fmoc-Lys(Cbz)-ψ[(*Z*)-CF=CH]-Lys(Cbz) [49] and Fmoc-Orn(Ns)-ψ[(*Z*)-CF=CH]-Nal were obtained [50].

**Scheme 17:**
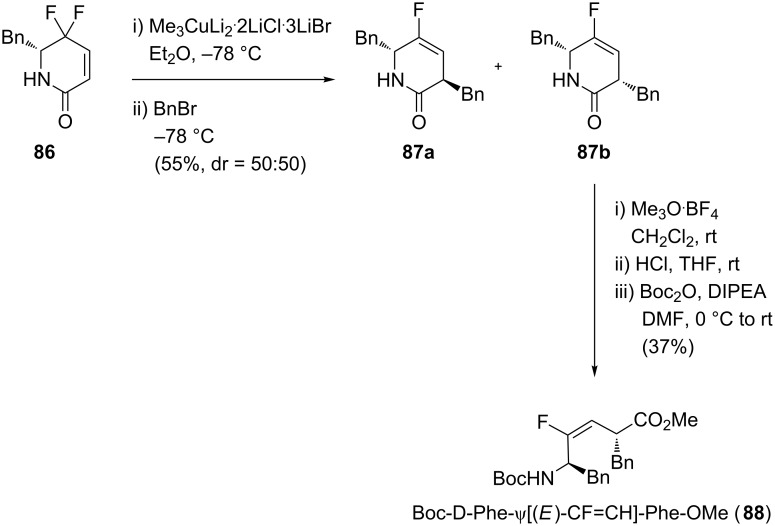
Synthesis of (*E*)-monofluoroalkene-based dipeptide isostere by Fujii and co-workers.

Fujii and co-workers also worked on the stereoselective synthesis of (*E*)-monofluoroalkenes (Scheme 17) [51]. To obtain a good selectivity towards the (*E*)-alkene, they relied on the copper-mediated reduction and the α-alkylation on the γ,γ-difluoro-α,β-unsaturated δ-lactam **86**. Unfortunately, the α-alkylation provided a mixture of diastereoisomers **87a** and **87b** which was separable by flash chromatography. The dipeptide isostere **88** was finally obtained after the opening of the lactam **87b** in acidic conditions and *N*-Boc protection.

#### Xaa-ψ[CF=C]-Pro

**Scheme 18:**
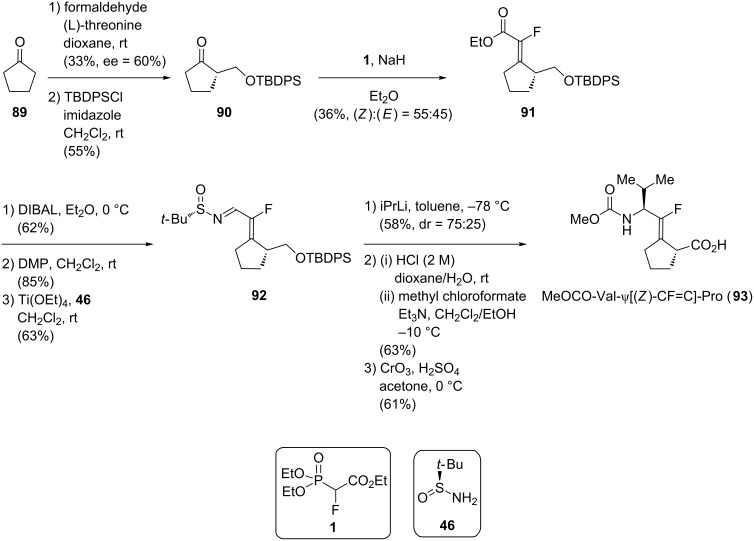
Diastereoselective synthesis of MeOCO-Val-ψ[(*Z*)-CF=C]-Pro isostere by Chang and co-workers.

The first asymmetric synthesis of Xaa-ψ[CF=C]-Pro was reported in 2012 by Chang’s group with the synthesis of MeOCO-Val-ψ[(*Z*)-CF=C]-Pro **93** (Scheme 18) [52]. Their synthesis started with a stereoselective aldol reaction using (L)-threonine to furnish a chiral β-hydroxy cyclopentanone **90**. A HWE olefination converted **90** into (*Z*)-monofluoroalkene **91** without any significant selectivity. The chiral Ellman’s sulfinylimine **92** was obtained in 3 steps. The diastereoselective addition of isopropyllithium was then possible to afford the (L)-Leu residue with a moderate selectivity (dr = 75:25). Further modifications (removal of the sulfinyl group and the silyl protecting group in acidic conditions, transformation of the amine in methyl carbamate and oxidation of the primary alcohol into the corresponding carboxylic acid) gave the final isostere **93**.

**Scheme 19:**
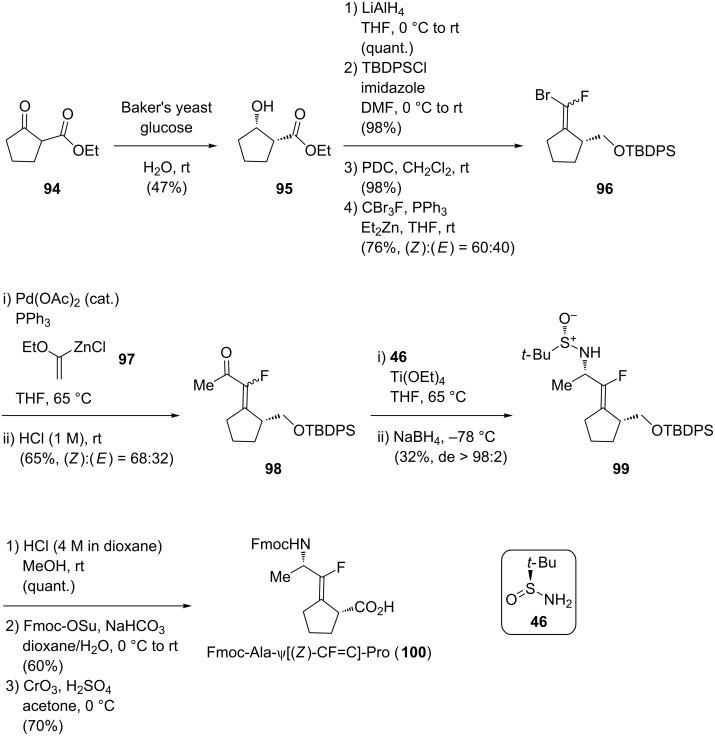
Asymmetric synthesis of Fmoc-Ala-ψ[(*Z*)-CF=C]-Pro by Pannecoucke and co-workers.

In 2013, Pannecoucke and co-workers proposed a new strategy based on a chemoenzymatic reduction of ethyl 2-oxocyclopentanecarboxylate (**94**) using Baker’s yeast to afford the corresponding chiral alcohol **95** (Scheme 19) [53]. Then, reduction of the ester into the primary alcohol, its selective protection by a silyl protecting group, oxidation of the secondary alcohol with pyridinium dichromate into the corresponding cyclopentanone derivative and subsequent olefination using CBr_3_F gave the monofluoroalkene **96** with a modest selectivity towards the (*Z*)-alkene. A Negishi coupling then gave alkene **98**. Stereoselective reductive amination using a chiral sulfonamide as chiral auxiliary afforded **99** (de > 98:2). Finally, group manipulations, i.e., deprotection of the amine, Fmoc reprotection and oxidation gave the isostere **100**.

**Scheme 20:**
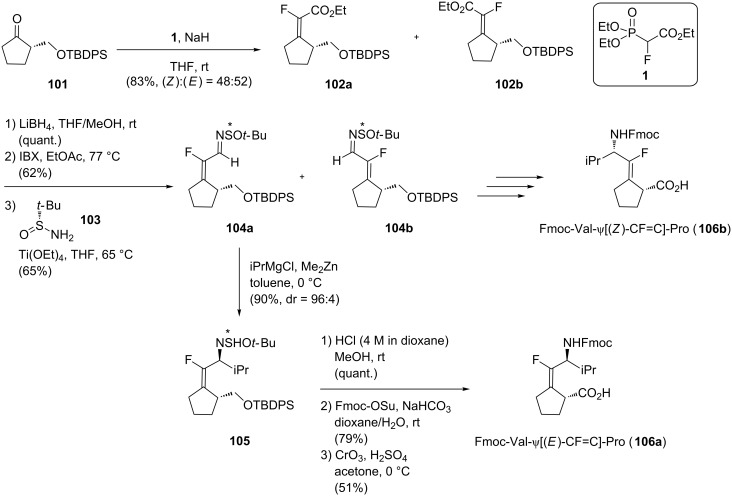
Synthesis of Fmoc-Val-ψ[(*E*)-CF=C]-Pro by Pannecoucke and co-workers.

Then, Pannecoucke’s group proposed a modified and more versatile approach where the monofluoroalkene **102** was synthesized by a HWE olefination of the chiral cyclopentanone **101** (Scheme 20) [54]. The resulting ester was converted into the aldehyde and β-fluoroenimine **104** was obtained using Ellman’s conditions. At this stage, the lateral chain of the N-terminal residue was added by an alkylation reaction using a Grignard reagent to give **105**. The last three steps (simultaneous deprotection of the amine and the alcohol in acidic conditions, Fmoc protection of the amine and oxidation of the alcohol into the corresponding carboxylic acid) led to the formation of three isosteres: Fmoc-Val-ψ[(*E*)-CF=C]-Pro (**106a**), Fmoc-Val-ψ[(*Z*)-CF=C]-Pro (**106b**) and Fmoc-Ala-ψ[(*Z*)-CF=C]-Pro.

### Applications

In this section, recent applications of monofluoroalkene-based dipeptide isosteres will be briefly described.

**Figure 4:**
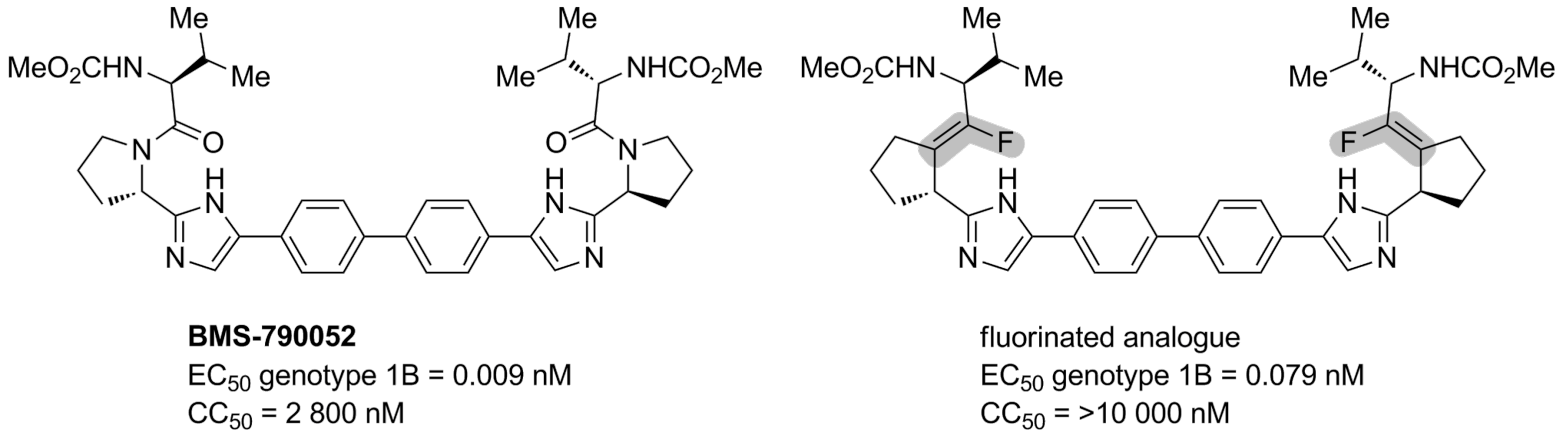
BMS-790052 and its fluorinated analogue.

Chang’s group used the Val-ψ[(*Z*)-CF=C]-Pro isostere (see Scheme 18) to synthesize a fluorinated analogue of BMS-790052, which is a promising inhibitor of the non-structural protein NS5A, an interesting target of the chronic hepatitis C virus [52]. The monofluoroalkene replaced the amide group, and the use of a dipeptide isostere containing a proline residue favoured a γ-turn substructure which is necessary for the interaction with the NS5A protein. This fluorinated peptide isostere showed activity in the picomolar range against one genotype and did not exhibit any cytotoxicity (Figure 4).

Pannecoucke and co-workers synthesized three heptapeptides, Gly-Gly-ψ[(*Z*)-CF=CH]-Phe-Ser-Phe-Arg-Phe-NH_2_, Gly-ψ[(*Z*)-CF=CH]-Gly-Phe-Ser-Phe-Arg-Phe-NH_2_ and Gly-ψ[(*E*)-CF=CH]-Gly-Phe-Ser-Phe-Arg-Phe-NH_2_ (see Scheme 7), representing the seven last amino acids of the neuropeptide 26Fra [34]. For the analogue containing the Gly-ψ[(*Z*)-CF=CH]-Phe isostere, epimerization was observed during last stages of the synthesis. The two diastereoisomers were separated after incorporation into the heptapeptide prior to biological studies, hence one had a D-Phe while the other had a L-Phe. The functional activity of the fluorinated mutants was evaluated by the calcium mobilizing response in GPR103-transfected cells. The peptides containing Gly-ψ[(*Z*)-CF=CH]-Gly or Gly-ψ[(*E*)-CF=CH]-Gly showed a higher activity than the non-fluorinated one, while an important decrease was observed for the peptides containing Gly-ψ[(*Z*)-CF=CH]-D-Phe and Gly-ψ[(*Z*)-CF=CH]-L-Phe (Table 3). These results underlined the importance of the Gly-Phe amide bond for the functional activity of the peptide. On the other hand, the fluorinated peptides showed a higher stability towards enzymatic degradation.

**Table 3:** Activity towards the calcium mobilizing response in GPR103-transfected cells of different 26FRa analogues.

entry	pseudopeptides	EC_50_ (nM)

1	Gly-Gly-Phe-Ser-Phe-Arg-Phe-NH_2_	(739 ± 149)
2	Gly-ψ[(*Z*)-CF=CH]-Gly-Phe-Ser-Phe-Arg-Phe-NH_2_	(618 ± 104)
3	Gly-ψ[(*E*)-CF=CH]-Gly-Phe-Ser-Phe-Arg-Phe-NH_2_	(538 ± 13)
4	Gly-Gly-ψ[(*Z*)-CF=CH]-D-Phe-Ser-Phe-Arg-Phe-NH_2_	6752
5	Gly-Gly-ψ[(*Z*)-CF=CH]-L-Phe-Ser-Phe-Arg-Phe-NH_2_	(1720 ± 1010)

Fujii’s group studied several applications of monofluoroalkene-based dipeptide isosteres. First, the affinity of the monofluoroalkene-based dipeptide isosteres Phe-ψ[(*Z*)-CF=CH]-Gly and Phe-ψ[(*E*)-CF=CH]-Gly for the peptide transporter PEPT1 was investigated (see Scheme 17) [51]. As the (*Z*)-monofluoroalkenes had a better bioactivity than the (*E*), the conclusion was that the transporter preferred the s-*trans* peptide bond. The *K*_i_ values obtained were also compared to the alkene analogues (Table 4).

**Table 4:** *K*_i_ values of Phe-Gly and analogues based on inhibition of [^3^H]Gly-Sar uptake by PEPT1 in Caco-2 cells.

entry	pseudopeptides	*K*_i_ (mM)

1	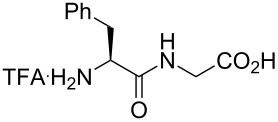	0.205
2	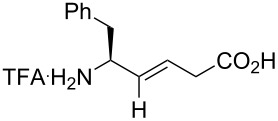	0.853
3	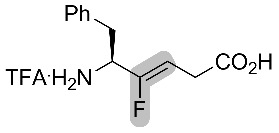	1.34
4	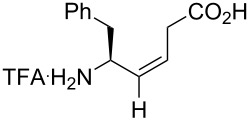	>10.0
5	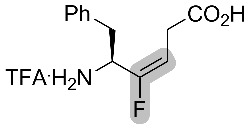	>10.0

**Figure 5:**
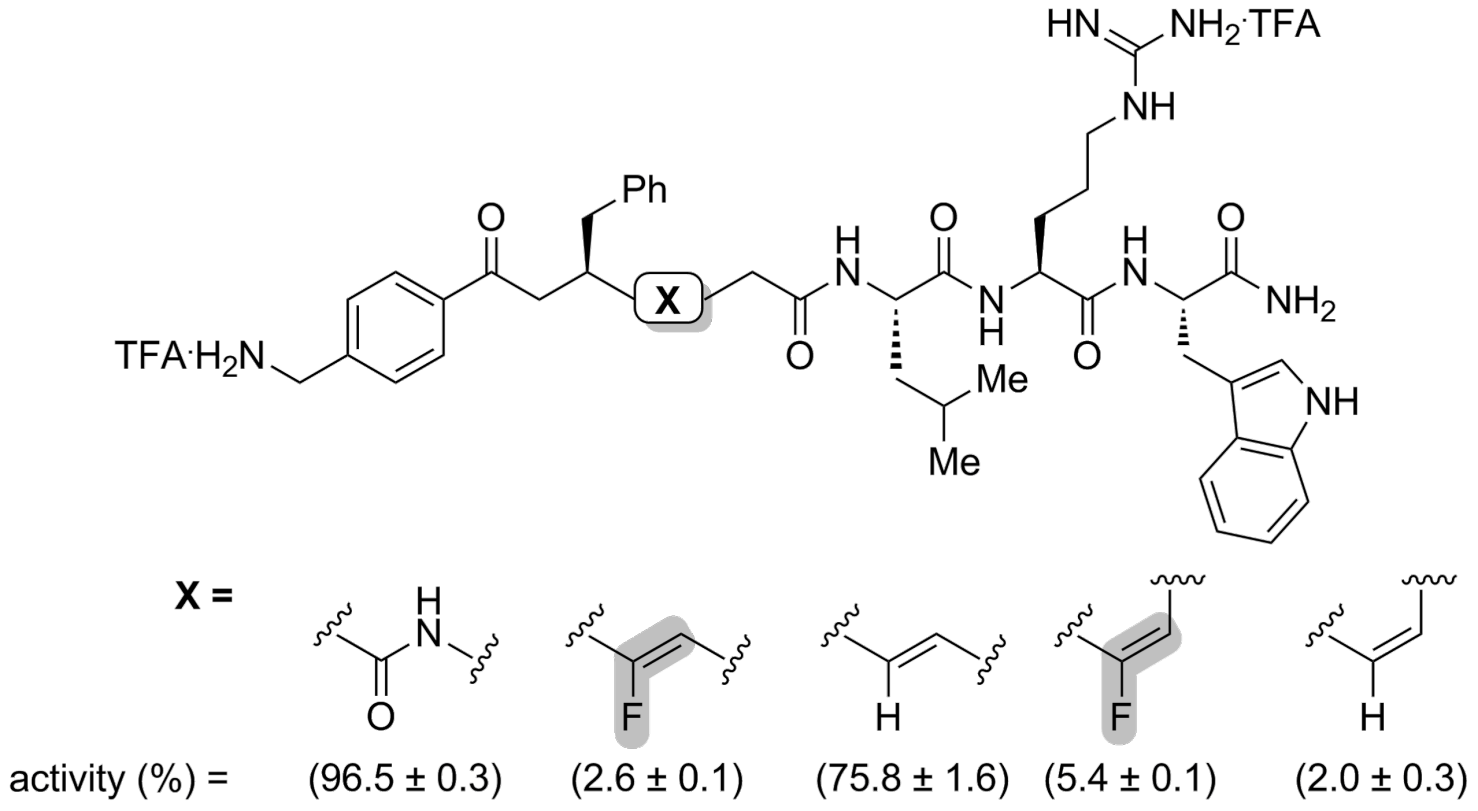
Bioactivities of pentapeptide analogues based on the relative maximum agonistic activity at 10 nM of the compound to 1 μM kisspeptin-10 (%). 100% = maximum agonistic activity at 1 μm kisspeptin-10.

Comparison between Phe-ψ[(*Z*)-CF=CH]-Gly and Phe-ψ[(*E*)-CF=CH]-Gly isosteres and their alkene analogues was also performed in an antagonist activity study towards GPR54. The fluorinated isosteres were incorporated into pentapeptides using Fmoc solid phase peptide synthesis (SPPS). Similar results as above were obtained in the sense that the activity of the s-*trans* peptide bond isostere was superior and that the fluorinated pseudopeptides were not more active than the natural peptide or the alkene-containing pseudopeptides (Figure 5) [55].

Fujii and co-workers also prepared, without isomerization or epimerization, cyclic pseudopeptides using Fmoc SPPS [48,50]. Biological studies were conducted on monofluoroalkene-containing analogues of FC131, which is a known antagonist of the chemokine receptor CXCR4. The latter has implications in cancer metastasis and HIV 1 infection. Anti-HIV 1 activity of the fluorinated antagonists showed an acceptable EC_50_ for the mutant containing Arg-ψ[(*Z*)-CF=CH]-Arg, while the one containing Arg-ψ[(*Z*)-CF=CH]-Nal (where NaI = L-3-(2-naphthyl)alanine) was not active (Table 5).

**Table 5:** Anti-HIV activities of FC131 and its fluorinated derivatives against three HIV strains.

entry	pseudopeptides	EC50 (μM)		
		
		NL4-3	IIIB	Ba-L

1	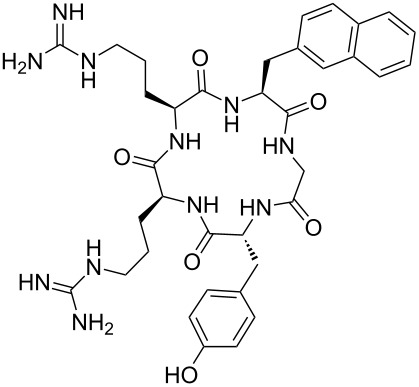	(0.014 ± 0.002)	(0.019 ± 0.003)	>10
2	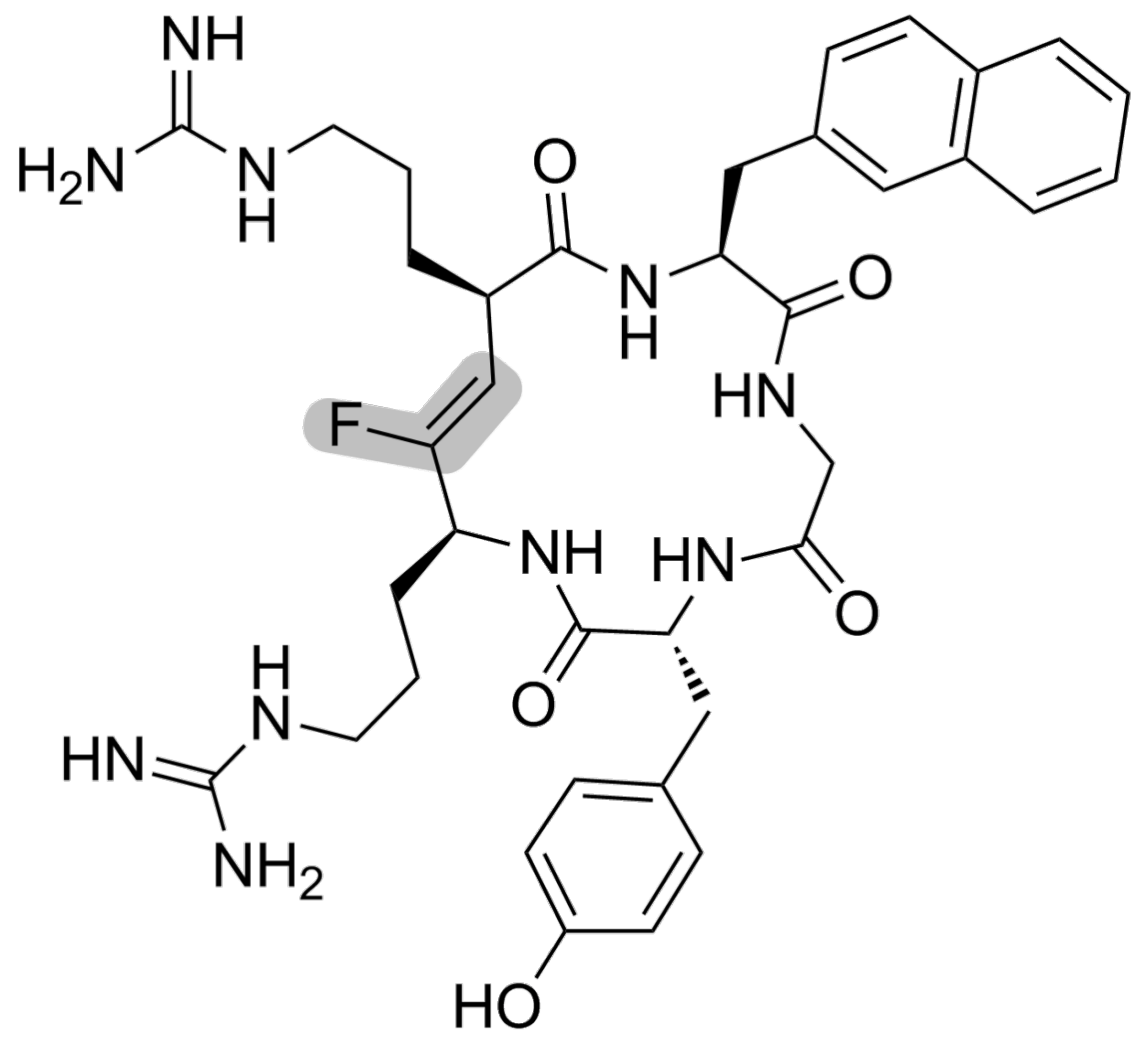	(0.332 ± 0.073)	(0.403 ± 0.051)	>10
3	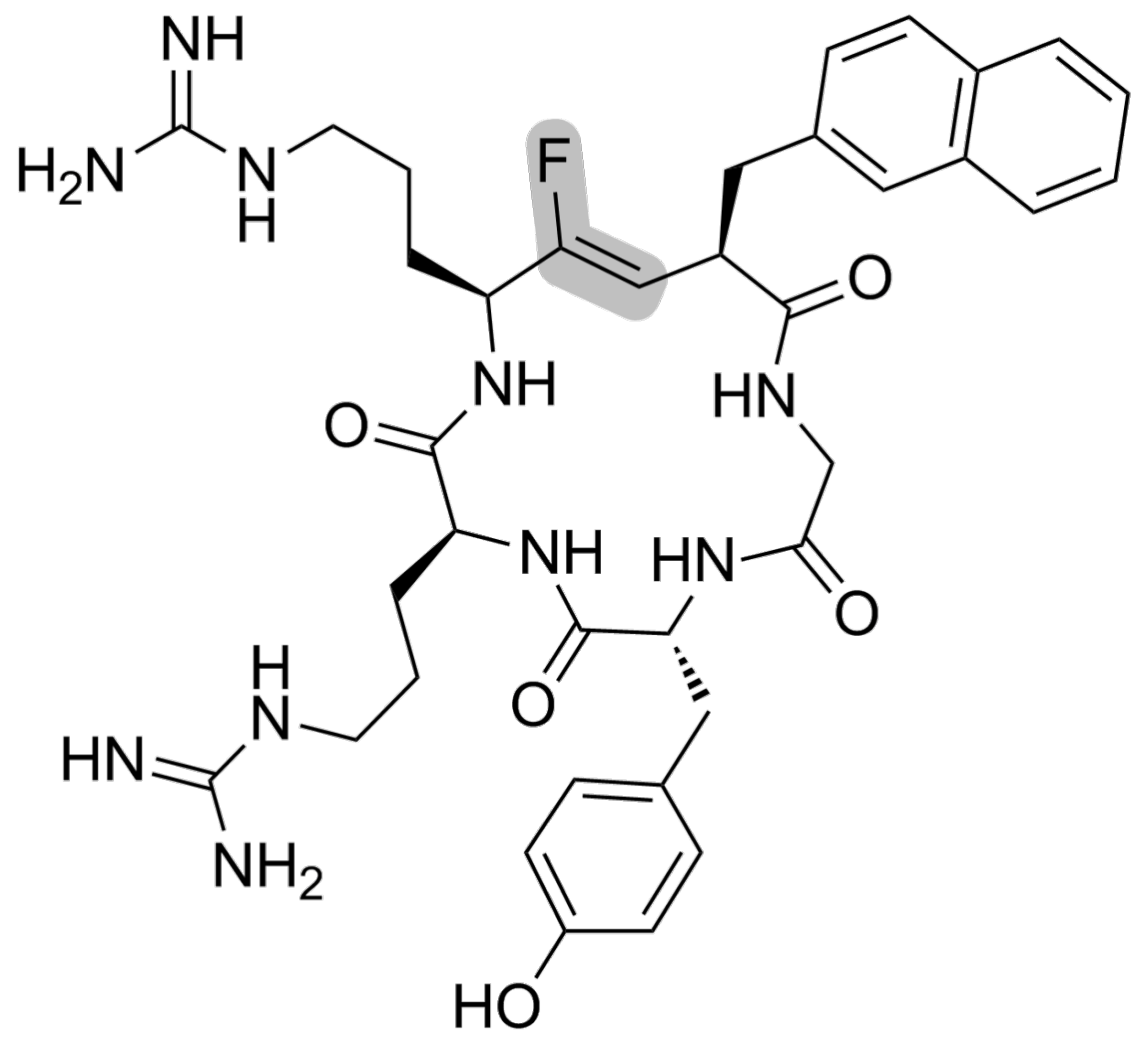	>10	>10	>10

Finally, incorporation at different positions of the monofluoroalkene-based dipeptide isostere Lys-ψ[(*Z*)-CF=CH]-Lys into a fusion inhibitory peptide active against HIV 1, SC29EK, was investigated [49]. Weak to moderate anti-HIV activity was observed for the fluorinated analogues, but the potency was always lower than for SC29EK. This suggested than the H-bonding behaviour was important for the activity (Table 6). Conformational studies of the fluorinated peptide using circular dichroism also showed that the incorporation of the monofluoroalkene did not perturb the formation of the secondary structure of the peptide, which was a α-helix.

**Table 6:** Anti-HIV activities of SC29EK and its fluorinated derivatives against three HIV strains. The number indicates the position of the dipeptide isostere.

entry	pseudopeptides	EC50 (nM)		
		
		NL4-3	IIIB	Ba-L

1	SC29EK	(2.2 ± 0.2)	(6.5 ± 0.9)	(1.9 ± 0.2)
2	SC29EK-6-Lys-ψ[(*Z*)-CF=CH]-Lys	(5220 ± 202)	>10 000	(5580 ± 1920)
3	SC29EK-13-Lys-ψ[(*Z*)-CF=CH]-Lys	(599 ± 96)	(3010 ± 554)	(600 ± 302)
4	SC29EK-20-Lys-ψ[(*Z*)-CF=CH]-Lys	(663 ± 242)	(2200 ± 712)	(527 ± 95)
5	SC29EK-27-Lys-ψ[(*Z*)-CF=CH]-Lys	(43 ± 7)	(237 ± 16)	(51 ± 7)

**Figure 6:**
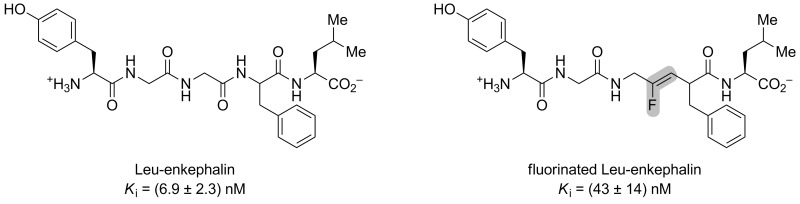
Structures and affinities of the Leu-enkephalin and its fluorinated analogue. The affinity towards DOPr was measured by competitive binding assays.

Dory and co-workers wanted to study the pentapeptide Leu-enkephaline, which can have analgesic properties when bounded to the DOPr receptor [36]. In order to study some derivative of the peptide, Fmoc-Gly-ψ[(*Z*-CF=CH]-Phe was synthesized (see Scheme 9). Using Fmoc SPPS, the fluorinated mutant was incorporated in the sequence of the Leu-enkephaline to obtain Tyr-Gly-Gly-ψ[(*Z*)-CF=CH]-Phe-Leu. The fluorinated Leu-enkephaline presented a 6-fold decreased binding affinity towards the DOPr receptor that the non-fluorinated analogue, showing that a hydrogen bond acceptor is necessary at this position of the peptide (Figure 6). The fluorinated peptide also showed higher lipophilicity, which can improve its pharmacokinetic properties.

**Figure 7:**
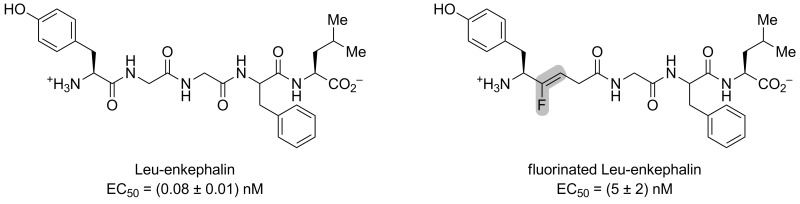
Activation of the opioid receptor DOPr by Leu-enkephaline and a fluorinated analogue.

Altman and co-workers also studied a fluorinated mutant of the Leu-enkephaline [41]. The isostere Boc-Tyr-ψ[(*Z*)-CF=CH]-Gly (see Scheme 12) was coupled to a tripeptide to afford Boc-Tyr-ψ[(*Z*)-CF=CH]-Gly-Gly-Phe-Leu. Then, the opioid activity was calculated towards the DOPr receptor and an EC_50_ in the nanomolar range was observed (Figure 7). Even if this value represented a 60-fold decrease compared to the non-fluorinated peptide, it showed that the fluorinated peptide binds to the receptor and that the amide bond at this position was not necessary. Thus, Boc-Tyr-ψ[(*Z*)-CF=CH]-Gly-Gly-Phe-Leu is shown to be a better isostere than Tyr-Gly-Gly-ψ[(*Z*)-CF=CH]-Phe-Leu for interactions with the DOPr receptor. The activity was also tested for the MOPr receptor, where it was lower than the Leu-enkephaline, and no activity was shown for the KOPr receptor.

## Conclusion

Different methodologies to synthesize monofluoroalkene-based dipeptide isosteres were developed since 2007. First, synthetic approaches to analogues in which there is no side chain or where the side chain stereochemistry is not controlled was discussed, either to obtain Gly-ψ[CF=CH]-Gly, Xaa-ψ[CF=CH]-Gly or Xaa-ψ[CF=C]-Pro. The synthesis of fluorinated isosteres with control of the stereochemistry at the side chain was then described, allowing the preparation of Gly-ψ[CF=CH]-Xaa, Xaa-ψ[CF=CH]-Gly, Xaa-ψ[CF=CH]-Xaa and Xaa-ψ[CF=C]-Pro. In both syntheses, control of the geometry of the fluoroalkene (i.e., *Z* vs *E*) was important. Finally, as the monofluoroalkene is of interest in medicinal chemistry as a non-hydrolyzable peptide bond isostere, some applications have been presented.
